# Defective α‐tectorin may involve tectorial membrane in familial Meniere disease

**DOI:** 10.1002/ctm2.829

**Published:** 2022-06-02

**Authors:** Pablo Roman‐Naranjo, Alberto M. Parra‐Perez, Alba Escalera‐Balsera, Andres Soto‐Varela, Alvaro Gallego‐Martinez, Ismael Aran, Nicolas Perez‐Fernandez, David Bächinger, Andreas H. Eckhard, Rocio Gonzalez‐Aguado, Lidia Frejo, Jose A. Lopez‐Escamez

**Affiliations:** ^1^ Otology & Neurotology Group CTS 495 Department of Genomic Medicine GENYO Centre for Genomics and Oncological Research: Pfizer/University of Granada/Andalusian Regional Government Granada Spain; ^2^ Sensorineural Pathology Programme Centro de Investigación Biomédica en Red en Enfermedades Raras Madrid Spain; ^3^ Department of Otolaryngology Instituto de Investigación Biosanitaria ibs.Granada Hospital Universitario Virgen de las Nieves Universidad de Granada Granada Spain; ^4^ Division of Otolaryngology Department of Surgery University of Granada Granada Spain; ^5^ Division of Otoneurology Department of Otorhinolaryngology Complexo Hospitalario Universitario Santiago de Compostela Spain; ^6^ Department of Surgery and Medical‐Surgical Specialities Universidade de Santiago de Compostela Santiago de Compostela Spain; ^7^ Department of Otolaryngology Complexo Hospitalario de Pontevedra Pontevedra Spain; ^8^ Department of Otorhinolaryngology Clinica Universidad de Navarra Madrid Spain; ^9^ Department of Otorhinolaryngology Head and Neck Surgery University Hospital Zurich, Zurich, Switzerland Zurich Switzerland; ^10^ Department of Otorhinolaryngology Hospital Universitario Marques de Valdecilla Cantabria Spain

Dear Editor

Although there have been considerable advances in recent years, the contribution of genetic factors to Meniere's disease (MD) is not yet fully understood. MD (OMIM 156000) is an inner ear disorder defined by episodes of vertigo associated with sensorineural hearing loss (SNHL) affecting low to medium frequencies, tinnitus, and aural fullness. Familial aggregation in MD has been described in 9–10% of European descendant population, showing an autosomal dominant inheritance pattern in most families.^1^ Despite familial MD displays extensive genetic heterogeneity,^2^ we recently observed multiple families carrying rare variants in genes encoding proteins involved in the structure of the hair cells stereocilia and their attachment to the tectorial membrane (TM): an enrichment of rare missense variants in the *OTOG* gene was found in 15 unrelated MD families^3^ and other 9 families showed rare heterozygous variants in the *MYO7A* gene.^4^ In this study, we have performed bioinformatic analyses in exome sequencing data obtained from patients of 77 families with MD to better understand the genetic underpinnings of the disease.

The results of the present study add new evidence to support the involvement of α‐tectorin in the pathophysiology of MD. We report four multicase MD families carrying rare missense heterozygous variants (F1–F3) and a short deletion (F4) in the coding region of the *TECTA* gene. Variants in this gene were also found in two additional families with one MD patient and relatives with partial syndromes carrying a missense heterozygous variant (F5) and a short deletion (F6) (Table 1). A briefcase report was made of each family to assess the genotype‐phenotype correlations (Supporting Results). Figure 1 and Supporting information Figure S1 shows the pedigrees and the pure‐tone audiograms, respectively, of the six families included in this study. Supporting information Table S1 shows a summary of the clinical information of familial MD patients carrying variants in the *TECTA* gene.

**TABLE 1:** Rare variants in the *TECTA* gene found in the studied familial MD cohort (N = 99)

Location	Protein change	Info	Exon	Domain	MD family	gnomAD_NFE_ MAF	gnomAD_MAX_ MAF	ACMG
11:121158016T > C	p.Val1494Ala	Missense	14	VWFD 4	F1 & F2	8.8 × 10^−5^	9.7 × 10^−5^ (AFR)	VUS
11:121152980G > C	p.Cys1402Ser	Missense	13	TIL	F3	1.5 × 10^−5^	4.8 × 10^−4^ (OTH)	VUS
11:121157956AC > A	p.Asn1474LysfsTer91	Deletion	14	–	F4	Novel	Novel	LP
11:121165368C > T	p.Pro1790Ser	Missense	17	–	F5	Novel	Novel	VUS
11:121189864GC > C	p.Gly2118ProfsTer22	Deletion	23	–	F6	0	2.4 × 10^−5^ (AFR)	LP

Reference sequence: NM_005422.2 (*TECTA*); ACMG, American College of Medical Genetics and Genomics; LP, Likely pathogenic; MAF, Minor allele frequency; MAX, Highest MAF value in gnomAD populations; NFE, Non‐Finish European; VUS, Variant of unknown significance.

**FIGURE 1:**
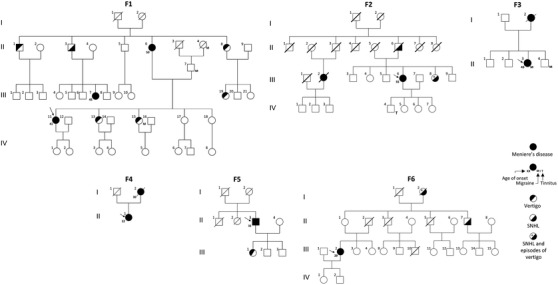
Pedigrees of the six families carrying rare variants and short deletions in the *TECTA* gene

The *TECTA* gene encodes α‐tectorin, a large protein that contains 10 functional domains divided into three major regions: (1) an entactin‐like (NIDO) region; (2) a larger middle region, the zonadhesin region containing a von Willebrand factor type C (VWFC) domain, four VWFD domains, and three trypsin inhibitor‐like cysteine‐rich (TIL) domains; and (3) the zona pellucida region.^5^ This protein is one of the main non‐collagenous proteins of the TM, an extracellular matrix that lies over the stereocilia of the sensory hair cells and mediates the deflection of the stereocilia and the gating of mechanotransduction channels. Variants in this gene are known to cause phenotypes of non‐syndromic autosomal hereditary hearing loss in humans.^6^ Furthermore, endolymphatic hydrops, a histopathological feature of MD, has been documented on Tecta^C1509G^ mouse model.^7^

The three variants in all multicase MD families were found clustered in the zonadhesin‐like region, whereas the two variants observed in the two families with partial syndromes were found close to the zona pellucida (Figure 2). To predict the impact of these rare variants on protein stability, the α‐tectorin domains were modelled using the AlphaFold2 method and assembled with the DEMO method (Figure 3 and Supporting Methods).

**FIGURE 2:**
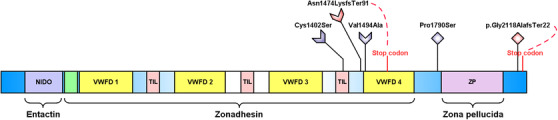
Variants distribution across the α‐tectorin domains. The α‐tectorin protein has several domains: the NIDO domain, the von Willebrand factor type C (VWFC: colored in green) and type D (VWFD) domains, the trypsin Inhibitor‐like cysteine‐rich (TIL) domain, and the zona‐pellucida (ZP) domains. Three rare missense variants (colored in blue) and two short deletions (colored in red) were found in four multicase MD families (arrow shaped) and two additional families with one MD patient and relatives with partial syndromes (diamond shaped). The position of stop codons generated by frameshift short deletions is indicated in red

**FIGURE 3:**
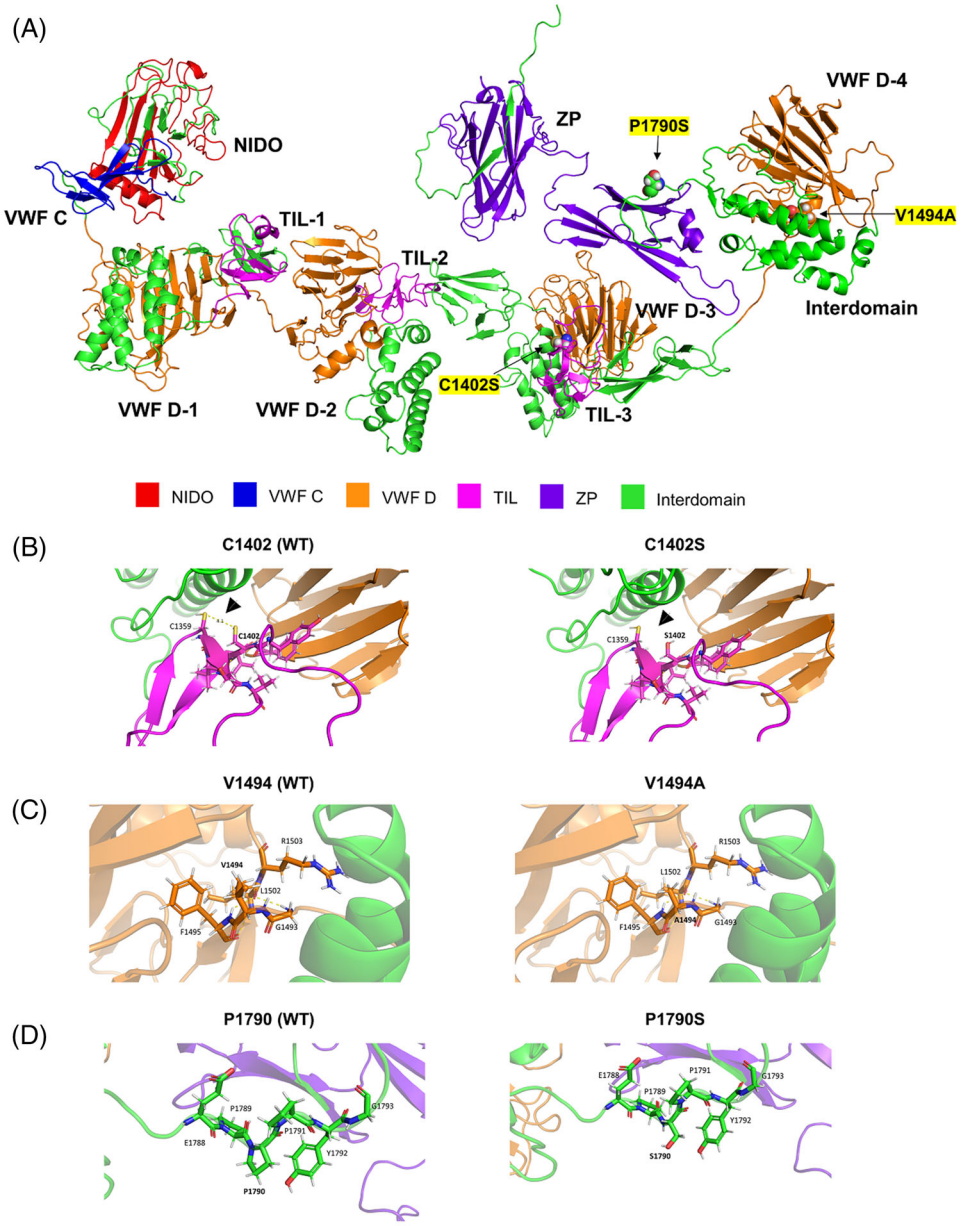
Predicted effect of variants on the α‐tectorin structure. (A) Mature α‐tectorin model showing the different domains that form the protein and the positions of the missense variants found in this study (indicated with black arrows). (B) TIL‐3 domain of WT α‐tectorin (left) and C1402S mutant (right). A disulfide bridge between C1359 and C1402 is predicted in the WT protein (indicated with an arrow). This disulfide bridge disappears in the mutated protein, where appears a weak polar interaction between C1359 and S1402. (C) VWF D‐4 domain of WT α‐tectorin (left) and V1494A mutant (right). (D) Inter‐domain upstream the ZP domain of α‐tectorin WT (left) and P1790S mutant (right). Yellow dashed lines represent polar interactions between residues within 4 Å from the mutated residue. Arrows show new or missing bonds. VWF D, Von Willebrand factor type D; ZP, zona pellucida

Of note, the variant p.Val1494Ala was found in F1, F2, and a sporadic case. The segregation of this variant was confirmed in F1, where it segregated in all three affected MD individuals and the father of III‐7, who only suffered from hearing loss (Figure 1). The missense variant p.Cys1402Ser found in F3 was classified as a variant of unknown significance (VUS). According to the protein model, the change of cysteine to serine at residue 1402 can lead to the breaking of the Cys1359‐Cys1402 disulphide bond with a destabilizing effect on α‐tectorin (Figure 3). In addition, the novel frame shift deletion in p.Asn1474LysfsTer91 found in F4, classified as likely pathogenic, resulted in a premature stop codon after 91 amino acids that generates a truncated protein of 1594 residues instead of 2155.

The two additional variants observed in the families with partial syndromes were classified as VUS. The novel variant p.Pro1790Ser was found in F5 segregating in two patients, the father with MD and his daughter with only vestibular symptoms. Finally, the short deletion p.Gly2118ProfsTer22 observed in F6 causes a premature stop codon at position 2139, generating a slightly shorter protein with a modified C‐terminal region and, in turn, an alteration of the glycosylphosphatidylinositol (GPI) anchorage signal located in this region. The α‐tectorin suffers a post‐translational modification in which it is tethered to the membrane via GPI.^8^ Thus, this frame shift deletion could be involved in an alteration of the TM during its formation by the modification of the GPI anchorage signal and leading to the clinical phenotype.

We hypothesized that these deletions and missense variants in the *TECTA* gene could change the TM micromechanics involved in the sound‐evoked motion of stereocilia causing hearing fluctuation in familial MD (Supporting Discussion). On the other hand, and unlike the hearing loss phenotype, the association between variants in α‐tectorin and vestibular dysfunction has not been established since most patients reported in the literature carrying variants in α‐tectorin do not show a vestibular phenotype. Nevertheless, some of these patients reported episodic vertigo or showed vestibular hyporeflexia.^9^ Furthermore, the expression of α‐tectorin in the vestibular system has been demonstrated in several mouse models, being detected in the saccule and the utricule.^10^ However, other genes or epigenetic factors that could modulate the vestibular phenotype in familial MD patients should be considered.

In conclusion, the results of this study seem to be in line with the results obtained in recent genetic studies about familial MD, where rare variants in *MYO7A* and *OTOG* gene were suggested to modify the stability or the interactions of different proteins in the apical surface of the sensory epithelia such as hair cells (stereocilia) or TM. In this study, the presence of rare missense variants and frame shift deletions in the *TECTA* gene in six unrelated families with MD suggests a role of this gene in the pathophysiology of the disease. However, because of the lack of a reliable association between α‐tectorin and vestibular function, we consider that additional variants and genes may contribute to the vestibular phenotype in MD.

## CONFLICT OF INTEREST

The authors declare that the research was conducted in the absence of any commercial or financial relationships that could be construed as a potential conflict of interest.

## Supporting information

Supporting Information
