# Supplementary material for: Defective α‐tectorin may involve tectorial membrane in familial Meniere disease
Source: Clin Transl Med. 2022 Jun 2;12(6):e829. doi: 10.1002/ctm2.829 (PMC9162437; doi:10.1002/ctm2.829)

SUPPORTING MATERIAL

Defective α-tectorin may involve tectorial membrane in familial Meniere disease

Contents

SUPPORTING METHODS 1
SUPPORTING RESULTS 6
SUPPORTING DISCUSSION 11
SUPPORTING REFERENCES 15
SUPPORTING TABLES 19
SUPPORTING FIGURES 25

CLINICAL AND TRANSLATIONAL MEDICINE

PABLO ROMAN-NARANJO, ALBERTO M. PARRA-PEREZ, ALBA ESCALERA-BALSERA, ANDRES SOTO-VARELA, ALVARO GALLEGO-MARTINEZ, ISMAEL ARAN, NICOLAS PEREZ-FERNANDEZ, DAVID BÄCHINGER, ANDREAS H. ECKHARD, ROCIO GONZALEZ-AGUADO, LIDIA FREJO, JOSE ANTONIO LOPEZ-ESCAMEZ

SUPPORTING METHODS

Ethics

The experimental protocol of this study was approved by the Institutional Review Board in all participating hospitals, and each participant signed a written informed consent before donating samples (Protocol PY20-00303). The study was carried out according to the principles of the Declaration of Helsinki revised in 2013 for investigation with human subjects.

Patient assessment, selection, and clinical phenotyping

MD patients were diagnosed and recruited from different Spanish hospitals within the Meniere's Disease Consortium (MeDiC). Patients were diagnosed according to the diagnostic criteria defined by the International Classification Committee for Vestibular Disorders of the Barany Society in 2015[1]. A complete audiological and vestibular assessment was performed, including magnetic resonance imaging in all cases, to rule out other vestibular diseases that could explain the phenotype. Pure-tone audiograms were retrieved to assess hearing loss since the onset of the disease. A total of 99 Spanish patients with familial MD over 18 years old from 77 different families were selected for exome sequencing.

DNA extraction and exome sequencing

Blood or saliva samples were collected to obtain DNA as previously described[2]. DNA samples were extracted with QIAamp DNA Mini Kit (Qiagen, Venlo, The Netherlands) and prepIT-L2P (DNA Genotek, Ottawa, Canada), respectively, following the manufacturer's protocols. DNA concentration and quality parameters were checked by Nanodrop (Thermofisher) and Qubit (Invitrogen) to assess that the samples reach the quality and concentration required for exome sequencing. Additionally, DNA integrity was verified by electrophoresis in a 2% agarose gel.

Exome sequencing was chosen for this study. All exomes were sequenced as previously described[3]. Exome libraries and coding regions were selected using the Agilent SureSelect XT v6 Exome kit (Agilent Technologies, Santa Clara, CA, USA). Sequencing was performed in a NovaSeq 6000 platform (Illumina) with a mean coverage of 100X.

Processing and dataset generation

All sequenced samples were aligned using the GRCh38/Hg38 reference genome with the maximal exact matches algorithm from Burrows-Wheeler Aligner. For variant calling, we followed standard recommended criteria from GATK. Exome reference alignment, base quality score recalibration, variant calling, and quality filtering pipeline was addressed using Sarek Nextflow pipeline (NF-ACore)[4]. Post-alignment processing was used to remove duplicated reads and the quality of the alignment itself was assessed[5], genetic variants were then called using the Haplotypecaller function from GATK. After the calling, we merged all the files to generate the MD variant dataset. We performed a variant quality filtering step using the variant quality score recalibration approach recommended by GATK. As a result, two variant call format files were generated, retrieving single nucleotide variants (SNVs) and short insertion and deletions from the sequenced exomes.

Annotation and prioritization strategy

To annotate the MD variant dataset, we used the Variant Effect Predictor (VEP, Ensembl). Then, we selected minor allele frequency (MAF) thresholds of 0.005 and 0.0005 to identify both autosomal-recessive and autosomal-dominant rare variants in the familial MD cohort, respectively, based on the data from a multi-ethnic study that assessed the pathogenicity of reported NSHL variants[6]. Allelic frequencies were retrieved for the non-Finish European (NFE) population from the Exome Aggregation Consortium (ExAC; N=32,299) and the Genome Aggregation Database (gnomAD; N=33,365) databases, and the Spanish population from the Collaborative Spanish Variant Server (CSVS; N=1,942) database[7]. Candidate variants were then classified

according to the American College of Medical Genetics (ACMG) and the Association for Molecular Pathology (AMP) guidelines[8], according to the specific guidelines for variant interpretation in genetic hearing loss[9]. Additionally, *in-silico* tools such as Sorting Intolerant From Tolerant (SIFT; SIFT<0.05), Polymorphism Phenotyping (Polyphen; Polyphen>0.446), Genomic Evolutionary Rate Profiling (GERP; GERP>2) or Combined Annotated Dependent Depletion (CADD; CADD>15) were used to prioritize and classify each variant according to its predicted pathogenicity.

Lastly, as an additional variant prioritization method, candidate genes carrying rare variants were associated with mammalian phenotypes using the Mouse Genome Database (http://www.informatics.jax.org). Similarly, the Human Phenotype Ontology Project (https://hpo.jax.org/app/) and the Online Mendelian Inheritance in Man (OMIM; https://omim.org/) databases were used to determine associations in humans between candidate genes and phenotypes.

Statistical analysis

European and Spanish databases such as ExAC, gnomAD, and CSVS were used as references to compare the observed MAF in familial MD. Odds ratios with 95% confidence interval were calculated for each single or set of variants. One-side p-values were corrected for multiple testing following the Bonferroni approach. A corrected p-value < 0.05 was considered statistically significant. The same approach was followed to assess if a combination of variants showed a significant association.

Candidate variant validation and representation

Regions with prioritized variants were visually inspected using the Integrative Genome Viewer software. Novel variants were validated using Sanger sequencing (Supporting Figure 2). Primers used for PCR (Supporting Table 2) were designed neighboring the regions flanking the variants using the Primer3 v4.1 (http://bioinfo.ut.ee/primer3/), Primer-Blast (https://www.ncbi.nlm.nih.gov/tools/primer-blast/), and the Oligoanalyzer

tool (https://eu.idtdna.com/calc/analyzer/). Candidate genes and variants were represented using Illustrator for Biological Sequences Version 1.0[10].

Hearing assessment and analysis

To analyze the time course of the hearing profile in familial MD cases with candidate variants, standard audiometric evaluations for air and bone conduction prompted by pure tones from 125 to 8,000Hz were retrieved from their clinical records.

Alpha-tectorin protein model

The effect of candidate variants on the α-tectorin protein structure was evaluated by protein modeling. The human α-tectorin mature amino acid sequence (from 23 to 2,091) was retrieved from Uniprot entry O75443, containing 10 functional domains. Structural models of the domains were generated using MODELLER (homology modeling)[11], Robetta-ab[12] (ab-initio modeling), and AlphaFold2[13] (ab-initio modeling) methods, and the best ones were assembled using the DEMO method[14]. Each of the generated models and the final assembly were validated using the structure validation algorithms MolProbity[15], Verify3D[16], ERRAT[17], ProSA-Web[18], and QMEANDisCo[19]. The *in-silico* model was used to predict the stability change (ΔΔG) of the α-tectorin produced by the candidate variants. Hence, we used DynaMut2[20], MAESTROweb[21], mCSM[22], PremPS[23] and CUPSAT[24] tools. Variants were classified as neutral when $-0.5 < \Delta\Delta G < 0.5$[25]. Finally, a prediction of Ca^{2+} ion binding sites was also performed to observe the possible effects of the variants on Ca^{2+} uptake with the metal ion-binding site prediction (MIB) method[26].

Variant data and protein structural model submission

All candidate variants in the *TECTA* gene have been submitted to the Clinvar database (http://www.ncbi.nlm.nih.gov/clinvar/). The accession numbers for these variants are as follow: SUB10993075, SUB10994469 and SUB10994413.

The α-tectorin structural model was submitted to the ModelArchive database (https://modelarchive.org/doi/10.5452/ma-xd6ic; Public access after publication. Temporary access code: mwnV7cphoi).

SUPPORTING RESULTS

Clinical description of families carrying variants in the TECTA gene

Family 1 (F1)

A family consisting of three women in two generations with the complete phenotype was selected for exome sequencing. In this family, six relatives had incomplete phenotypes: five relatives (four women and one man) with recurrent vertigo and one man with SNHL. Additionally, three relatives (two women and one man) also suffered from migraine.

The index case (III-11) was a woman suffering from right SNHL with episodes of vertigo since 2003 when she was 41-year-old. In 2007, the hearing loss evolved to bilateral SNHL. Her mother (II-6), who is currently 84 years old, was diagnosed with left-sided MD when she was 50 years old. In 2018, the last time she was examined, the patient presented bilateral hearing loss with a normal gain in the video head impulse test (vHIT). Finally, the relative III-7 was diagnosed with bilateral MD in 2010. Currently, III-7 is 56 years old and, when she was evaluated in 2018, she did not show hearing nor vestibular symptoms, showing normal hearing and vestibulo-ocular reflexes.

Family 2 (F2)

This family consists of two female cousins with MD in one generation (III-2 and III-6) where only III-6 (index patient) was available for exome sequencing. III-6 is a 70-year-old woman suffering from unilateral (left ear) hearing loss since she was 56. The disease progressed from unilateral to bilateral MD when she was 62 years old, suffering from that moment mainly vestibular symptoms. Some of her relatives had partial syndromes: her father (II-6) suffered from tinnitus, a trait that is also present in her son (IV-4). In addition, one of her sisters (II-8) presented SNHL and vertigo episodes who did not meet the diagnostic criteria for MD.

Family 3 (F3)

The third family consists of two relatives with MD, the mother (I-2) and her daughter (II-3), only being available for exome sequencing II-3. There are no intermediate phenotypes in this family, neither hearing loss, nor vertigo or tinnitus. Two brothers of II-3 have a history of high blood pressure. Additionally, one of them was diagnosed with cardiac valvulopathy and the other with psoriasis.

The index case (II-3) is currently a 56-year-old woman suffering from fluctuating hearing loss and tinnitus in her right ear since 2007 when she was 42. She experienced her first vertigo episode associated with fluctuating hearing loss at 45 years old. During the next 5 months, she suffered from 8 episodes of vertigo, 4 of them lasting more than two hours. Right canal paresis (62%) was evidenced by bithermal caloric testing. The patient started treatment with acetazolamide (250-500 mg daily) without any improvement in her symptoms. Next, two intratympanic instillations of dexamethasone (23mg/ml) were administrated in the right ear, with an improvement in control of vertigo. In a 10-year-follow-up, this patient suffered from 4 episodes of vertigo. Her mother (I-2) suffered from left-sided MD, but it progressed from unilateral to bilateral MD with severe hearing loss at the end of the disease. This patient used a combination of trimetazidine and vestibular sedatives to control vertigo attacks.

Family 4 (F4)

A fourth family consisting of two relatives with MD in two generations (mother and daughter) was studied. Only the daughter (II-1, index patient) was available for exome sequencing. Her mother, who died at the age of 39, suffered from unilateral MD since her thirties.

II-1 is currently a 79-year-old woman suffering from bilateral MD. At the age of 22, the disease started as unilateral, affecting her right ear. We could not assess the hearing loss progression in this ear since the first audiogram we could recover was from 1972, when the patient already suffered from severe SNHL affecting all frequencies. Over the

years, the disease progressed affecting both ears, estimating the onset of the left hearing loss between the ages of 40 and 50. Hearing loss progressed in both ears showing a flat-type audiogram at the age of 77. The vestibular assessment was performed at 76-year-old by vHIT testing, showing a bilateral vestibular hypofunction. Due to a tympanostomy tube, caloric testing could not be performed. In this same year, the patient got a cochlear implant in her right ear.

Family 5 (F5)

A family consisting of a man with MD (II-3; index patient) and his daughter (III-1) with partial syndromes. II-3 is a 79-year-old man suffering from bilateral MD since he was 31. We could not evaluate the hearing progression on this patient since the first audiogram we were able to retrieve was from 2005, when he was 62 and the disease had progressed for 31 years. At that time, hearing loss was already affecting all frequencies in both ears. His daughter, currently 52 years old, reported episodes of vertigo accompanied by tinnitus and headache during the last 5 years. However, she did not show a hearing loss. None of the other relatives reported MD nor partial syndromes: II-1 and III-2 suffered from high blood pressure and II-2 suffered from diabetes mellitus type 2.

Family 6 (F6)

The last family consisted of a woman with MD (IV-3) and two other relatives with partial syndromes (a woman (I-1) and a man (II-7)). The index patient (IV-3) is a 55-year-old woman suffering from recurrent vertigo associated with tinnitus and aural fullness (left ear) since she was 29. This patient suffers from profound unilateral hearing loss affecting all frequencies since the first years of the disease. According to the familial pedigree, these traits (i.e., hearing loss or vertigo) were exclusively identified on the maternal side of the proband: her maternal grandmother (I-2) suffered from unknown etiology hearing loss and vertigo and her uncle (II-7) only suffered from hearing loss.

Sporadic patient

We have also identified a patient (woman; 75 years old) with sporadic MD carrying the same missense variant in the TECTA gene as F1 and F2 patients. This patient reported her first episode of vertigo in 2008 at 62, when she had two prolonged episodes of vertigo, referring to a sensation of spinning motion, right-sided hearing loss, and vegetative symptoms. One week after her second vertigo episode, the audiogram showed normal thresholds except for a slight drop in high frequencies.

Six years later, the patient returned to the hospital reporting a new episode of vertigo, beginning with a sensation of spinning motion followed by tinnitus and hearing loss in her right ear. At that time, the audiogram showed a moderate to severe SNHL in her right ear involving all frequencies. During that year, the patient reported new episodes of vertigo. Caloric testing was conducted, showing normal results. Vertigo attacks were controlled with steroids and betahistine.

Three years later, when she was 71, the patient reported multiple episodes of vertigo lasting several hours with worsening hearing loss and aural fullness. The patient followed her treatment of betahistine and a salt-reduced diet was recommended. During the last four years, the patient has not reported any vestibular symptoms, although she has referred tinnitus in her right ear.

Protein modeling

The α-tectorin protein model was obtained by modeling the protein domains using the AlphaFold2 method and assembled with the DEMO method (Main Figure 3). According to the geometrical validation results (Supporting Table 3), we have obtained a reliable model compared to structures solved by experimental techniques at the geometric level. This model was used to predict the impact of SNVs on protein stability and Ca^{2+} binding sites.

p.Cys1402Ser (Cys1380Ser, in the mature protein without the signal peptide and the final propeptide), p.Val1494Ala (Val1472Ala), and p.Pro1790Ser (Pro1768Ser) variants were predicted *in-silico* to change the overall stability of the α-tectorin (Supporting Table 4). Consistent with the used methods, variants p.Cys1402Ser found in F3 and p.Val1494Ala found in F1 and F2 were classified as destabilizing variants. In contrast, most of the used predictors classified the novel variant p.Pro1790Ser found in F5 as a neutral variant according to the predicted perturbation on protein stability. Four probable Ca^{2+} binding sites were predicted in the α-tectorin VWFD domain where the p.Pro1790Ser is located, however, this variant appears to not affect them (Supporting Table 5 and Supporting Figure 3).

SUPPORTING DISCUSSION

Our findings support that rare variations in the *TECTA* gene may affect the stability of the protein α-tectorin in patients with familial Meniere disease (MD) suggesting a novel mechanism involving the tectorial membrane (TM) in this disease. This membrane, which is composed of a mixture of collagenous and non-collagenous proteins, plays a critical role in the process of hearing mediating the mechanical stimulation of cochlear hair cells[27]. Thus, variants in these non-collagenous proteins of the TM, namely otogelin (*OTOG*), otogelin-like (*OTOGL*), α-tectorin (*TECTA*), β-tectorin (*TECTB*), and the carcinoembryonic antigen cell adhesion molecule 16 (*CEACAM16*), may result in different types of autosomal dominant or autosomal recessive hearing loss in humans: DFNB18B[28], DFNB84B[29], DFNA8/12[30], DNFB21[31], DFNA4B[32] or DFNB113[33]. In this study, we have identified 3 rare missense variants and 2 deletions in the *TECTA* gene in 4 multicase MD families and 2 families with MD and partial syndromes by exome sequencing. Of note, one of these rare variants (p.Val1494Ala) was found in two unrelated families segregating the MD phenotype, and two deletions generating a truncated form of the α-tectorin protein in other two families. These variants in α-tectorin could change the TM stability, disturbing the sound-evoked motion of hair cell stereocilia in familial MD.

The *TECTA* gene encodes the α-tectorin protein, one of the major non-collagenous proteins of the TM[27]. Although there is little evidence, several molecular models involving α-tectorin, β-tectorin, and CEACAM16 have been proposed for the formation of the striated-sheet matrix, strands of filaments that organize the collagen fibers in the TM[34,35]. Both, α- and β-tectorin contain a ZP region, a polymerization domain that could mediate the formation of either homomeric or heteromeric filaments crosslinked by CEACAM16. Studies in mice lacking functional α-tectorin (Tecta$^{\Delta ENT/\Delta ENT}$) showed a TM absent of striated-sheet matrix and completely detached from the organ of Corti[36]. Furthermore,

several models carrying heterozygous variants in different Tecta domains were generated as models from human *TECTA* variants. These variants disrupted the structure of the covernet fibrils, the marginal band, the Hensen's stripe, and the Kimura's membrane[37,38]. The structural consequences of variants in the *TECTA* gene showed in this study are expected to be milder compared to the phenotype showed in the Tecta$^{\Delta ENT/\Delta ENT}$ mice model, resembling more the phenotypes exhibited by those mice models carrying heterozygous variants in Tecta.

In a recent article, Bullen *et al*. identified the age-related degradation of the tectorial membrane as a potential contributor to hearing loss in mice and humans[39]. Progressively with age, a decrease in the density of the core of the TM was observed. In mice, a progressive degeneration of the TM was first apparent in the apical coil from about 12–18 months then proceeded with age into the basal coil by 24–30 months. Mainly, this degeneration is due to the progressive loss of tectorins (TECTA and TECTB), proteins that are produced only for a short time during early development[40].

According to the *Tecta* mouse models, it is plausible that human also may have a progressive loss of tectorins with age. Our hypothesis is that MD patients with rare variants or deletions in *TECTA* may have an accelerated degradation of the TM. This loss of tectorins would be age-dependent and should be associated with a triggering factor that would increase endolymphatic pressure (e.g., acoustic trauma). So, the risk to develop MD in carriers of *TECTA* variants would be higher in adults than in children, causing late onset hearing loss in these patients. A triggering factor could, in turn, influence the age of onset or the hearing loss laterality. The fluctuating symptoms could be mediated by changes in the endolymph pressure, inducing the coupling/decoupling of TM and stereocilia.

The SNVs described in this study were investigated at the functional level according to the predicted α-tectorin protein model. The variant p.Val1494Ala was found in F1, F2 and a sporadic case of our sporadic MD cohort, being classified as VUS according to the

ACMG/AMP criteria. Although this variant was located in the VWFD domain, a domain that can bind Ca2+, the variant p.Val1494Ala seems to have no effect in the Ca2+ binding sites predicted using the protein model structure. In addition, based on the protein stability predictors, this variant produces a slightly destabilizing effect on α-tectorin. The missense variant p.Cys1402Ser found in F3 was classified as a VUS according to the ACMG/AMP guidelines. The change of cysteine to serine at residue 1402 can lead to the breaking of the Cys1359-Cys1402 disulfide bond. The suggested disulfide bond disruption could destabilize the striated-sheet matrix structure of the TM, causing progressive hearing loss[41].

A novel frameshift deletion p.Asn1474LysfsTer91 was found in F4, resulting in the absence of the fourth VWFD domain and the ZP domain. ZP and VWFD domains have been suggested to be involved in the TM matrix assembly through the formation of homomeric filaments of α-tectorin or heteromeric filaments with β-tectorin[42,43]. In addition, the TM has been suggested as a reservoir for Ca2+ cations, which are needed to control the mechanotransduction channel in the stereocilia of hair cells[44]. Apparently, behind this function are the VFWD domains of α-tectorin and otogelin proteins, which can bind Ca2+ ions[45]. Thus, the short deletion found in F4 could potentially decrease Ca2+ uptake by the VWFD domain and prevent the formation of α-tectorin homodimers and α-tectorin/β-tectorin heterodimers.

The novel variant p.Pro1790Ser in F5 was classified as a neutral variant in terms of protein stability perturbation by 4 out of 6 prediction tools. Nevertheless, we cannot discard a deleterious effect of this variant for a reason different from stability. In fact, this variant, which affects the ZA-ZP interdomain of α-tectorin, is only one amino acid residue downstream of the p.Pro1791Arg variant, firstly described by Hildebrand et al. in an American family suffering from prelingual mid-frequency SNHL[46]. Later, this variant was also found in a man suffering from progressive, postlingual SNHL[47].

In general, we observed a trend towards postlingual hearing loss worsening with age involving all frequencies. Conversely, we did not identify a different audiometric profile according to the region where the variants were localized, nor the cookie-bite/U-shaped audiogram typically observed in patients carrying recessive mutations in *TECTA*[48–50]. Interestingly, the patients carrying frameshift deletions in this study (i.e., F4 & F6) presented an earlier age of onset compared to those patients carrying rare missense variants, suggesting a more significant alteration in the TM matrix that could correlate to an earlier occurrence of the phenotype.

SUPPORTING REFERENCES

1. Lopez-Escamez JA, Carey J, Chung WH, et al. Diagnostic criteria for Menière's disease. *J Vestib Res Equilib Orientat*. 2015;25(1):1-7. doi:10.3233/VES-150549

2. Szczepek AJ, Frejo L, Vona B, et al. Recommendations on Collecting and Storing Samples for Genetic Studies in Hearing and Tinnitus Research. *Ear Hear*. 2019;40(2):219-226. doi:10.1097/AUD.0000000000000614

3. Roman-Naranjo P, Moleon MDC, Aran I, et al. Rare coding variants involving MYO7A and other genes encoding stereocilia link proteins in familial meniere disease. *Hear Res*. 2021;409:108329. doi:10.1016/j.heares.2021.108329

4. Garcia M, Juhos S, Larsson M, et al. Sarek: A portable workflow for whole-genome sequencing analysis of germline and somatic variants. *F1000Research*. 2020;9:63. doi:10.12688/f1000research.16665.2

5. McKenna A, Hanna M, Banks E, et al. The Genome Analysis Toolkit: a MapReduce framework for analyzing next-generation DNA sequencing data. *Genome Res*. 2010;20(9):1297-1303. doi:10.1101/gr.107524.110

6. Shearer AE, Eppsteiner RW, Booth KT, et al. Utilizing ethnic-specific differences in minor allele frequency to recategorize reported pathogenic deafness variants. *Am J Hum Genet*. 2014;95(4):445-453. doi:10.1016/j.ajhg.2014.09.001

7. Peña-Chilet M, Roldán G, Perez-Florido J, et al. CSVS, a crowdsourcing database of the Spanish population genetic variability. *Nucleic Acids Res*. 2021;49(D1):D1130-D1137. doi:10.1093/nar/gkaa794

8. Richards S, Aziz N, Bale S, et al. Standards and guidelines for the interpretation of sequence variants: a joint consensus recommendation of the American College of Medical Genetics and Genomics and the Association for Molecular Pathology. *Genet Med Off J Am Coll Med Genet*. 2015;17(5):405-424. doi:10.1038/gim.2015.30

9. Oza AM, DiStefano MT, Hemphill SE, et al. Expert specification of the ACMG/AMP variant interpretation guidelines for genetic hearing loss. *Hum Mutat*. 2018;39(11):1593-1613. doi:10.1002/humu.23630

10. Liu W, Xie Y, Ma J, et al. IBS: an illustrator for the presentation and visualization of biological sequences. *Bioinforma Oxf Engl*. 2015;31(20):3359-3361. doi:10.1093/bioinformatics/btv362

11. Webb B, Sali A. Protein Structure Modeling with MODELLER. *Methods Mol Biol Clifton NJ*. 2021;2199:239-255. doi:10.1007/978-1-0716-0892-0_14

12. Kim DE, Chivian D, Baker D. Protein structure prediction and analysis using the Robetta server. *Nucleic Acids Res*. 2004;32(Web Server issue):W526-531. doi:10.1093/nar/gkh468

13. Jumper J, Evans R, Pritzel A, et al. Highly accurate protein structure prediction with AlphaFold. *Nature*. 2021;596(7873):583-589. doi:10.1038/s41586-021-03819-2

14. Zhou X, Hu J, Zhang C, Zhang G, Zhang Y. Assembling multidomain protein structures through analogous global structural alignments. *Proc Natl Acad Sci U S A*. 2019;116(32):15930-15938. doi:10.1073/pnas.1905068116

15. Williams CJ, Headd JJ, Moriarty NW, et al. MolProbity: More and better reference data for improved all-atom structure validation. *Protein Sci Publ Protein Soc*. 2018;27(1):293-315. doi:10.1002/pro.3330

16. Eisenberg D, Lüthy R, Bowie JU. VERIFY3D: assessment of protein models with three-dimensional profiles. *Methods Enzymol*. 1997;277:396-404. doi:10.1016/s0076-6879(97)77022-8

17. Colovos C, Yeates TO. Verification of protein structures: patterns of nonbonded atomic interactions. *Protein Sci Publ Protein Soc*. 1993;2(9):1511-1519. doi:10.1002/pro.5560020916

18. Wiederstein M, Sippl MJ. ProSA-web: interactive web service for the recognition of errors in three-dimensional structures of proteins. *Nucleic Acids Res*. 2007;35(Web Server issue):W407-410. doi:10.1093/nar/gkm290

19. Studer G, Rempfer C, Waterhouse AM, Gumienny R, Haas J, Schwede T. QMEANDisCo-distance constraints applied on model quality estimation. *Bioinforma Oxf Engl*. 2020;36(6):1765-1771. doi:10.1093/bioinformatics/btz828

20. Rodrigues CHM, Pires DEV, Ascher DB. DynaMut2: Assessing changes in stability and flexibility upon single and multiple point missense mutations. *Protein Sci Publ Protein Soc*. 2021;30(1):60-69. doi:10.1002/pro.3942

21. Laimer J, Hiebl-Flach J, Lengauer D, Lackner P. MAESTROweb: a web server for structure-based protein stability prediction. *Bioinforma Oxf Engl*. 2016;32(9):1414-1416. doi:10.1093/bioinformatics/btv769

22. Pires DEV, Ascher DB, Blundell TL. mCSM: predicting the effects of mutations in proteins using graph-based signatures. *Bioinformatics*. 2014;30(3):335-342. doi:10.1093/bioinformatics/btt691

23. Chen Y, Lu H, Zhang N, Zhu Z, Wang S, Li M. PremPS: Predicting the impact of missense mutations on protein stability. *PLoS Comput Biol*. 2020;16(12):e1008543. doi:10.1371/journal.pcbi.1008543

24. Parthiban V, Gromiha MM, Schomburg D. CUPSAT: prediction of protein stability upon point mutations. *Nucleic Acids Res*. 2006;34(Web Server issue):W239-242. doi:10.1093/nar/gkl190

25. Pancotti C, Benevenuta S, Birolo G, et al. Predicting protein stability changes upon single-point mutation: a thorough comparison of the available tools on a new dataset. *Brief Bioinform*. Published online January 11, 2022:bbab555. doi:10.1093/bib/bbab555

26. Lin YF, Cheng CW, Shih CS, Hwang JK, Yu CS, Lu CH. MIB: Metal Ion-Binding Site Prediction and Docking Server. *J Chem Inf Model*. 2016;56(12):2287-2291. doi:10.1021/acs.jcim.6b00407

27. Goodyear RJ, Richardson GP. Structure, Function, and Development of the Tectorial Membrane: An Extracellular Matrix Essential for Hearing. *Curr Top Dev Biol*. 2018;130:217-244. doi:10.1016/bs.ctdb.2018.02.006

28. Schraders M, Ruiz-Palmero L, Kalay E, et al. Mutations of the Gene Encoding Otogelin Are a Cause of Autosomal-Recessive Nonsyndromic Moderate Hearing Impairment. *Am J Hum Genet*. 2012;91(5):883-889. doi:10.1016/j.ajhg.2012.09.012

29. Yariz KO, Duman D, Zazo Seco C, et al. Mutations in OTOGL, encoding the inner ear protein otogelin-like, cause moderate sensorineural hearing loss. *Am J Hum Genet*. 2012;91(5):872-882. doi:10.1016/j.ajhg.2012.09.011

30. Verhoeven K, Van Laer L, Kirschhofer K, et al. Mutations in the human alpha-tectorin gene cause autosomal dominant non-syndromic hearing impairment. *Nat Genet*. 1998;19(1):60-62. doi:10.1038/ng0598-60

31. Mustapha M, Weil D, Chardenoux S, et al. An alpha-tectorin gene defect causes a newly identified autosomal recessive form of sensorineural pre-lingual non-syndromic deafness, DFNB21. *Hum Mol Genet*. 1999;8(3):409-412. doi:10.1093/hmg/8.3.409

32. Zheng J, Miller KK, Yang T, et al. Carcinoembryonic antigen-related cell adhesion molecule 16 interacts with alpha-tectorin and is mutated in autosomal dominant hearing loss (DFNA4). *Proc Natl Acad Sci U S A*. 2011;108(10):4218-4223. doi:10.1073/pnas.1005842108

33. Booth KT, Kahrizi K, Najmabadi H, Azaiez H, Smith RJ. Old gene, new phenotype: splice-altering variants in CEACAM16 cause recessive non-syndromic hearing impairment. *J Med Genet*. 2018;55(8):555-560. doi:10.1136/jmedgenet-2018-105349

34. Kammerer R, Rüttiger L, Riesenberg R, et al. Loss of mammal-specific tectorial membrane component carcinoembryonic antigen cell adhesion molecule 16 (CEACAM16) leads to hearing impairment at low and high frequencies. *J Biol Chem*. 2012;287(26):21584-21598. doi:10.1074/jbc.M111.320481

35. Cheatham MA, Goodyear RJ, Homma K, et al. Loss of the tectorial membrane protein CEACAM16 enhances spontaneous, stimulus-frequency, and transiently evoked otoacoustic emissions. *J Neurosci Off J Soc Neurosci*. 2014;34(31):10325-10338. doi:10.1523/JNEUROSCI.1256-14.2014

36. Legan PK, Lukashkina VA, Goodyear RJ, Kössi M, Russell IJ, Richardson GP. A targeted deletion in alpha-tectorin reveals that the tectorial membrane is required for the gain and timing of cochlear feedback. *Neuron*. 2000;28(1):273-285. doi:10.1016/s0896-6273(00)00102-1

37. Xia A, Gao SS, Yuan T, et al. Deficient forward transduction and enhanced reverse transduction in the alpha tectorin C1509G human hearing loss mutation. *Dis Model Mech*. 2010;3(3-4):209-223. doi:10.1242/dmm.004135

38. Legan PK, Goodyear RJ, Morín M, et al. Three deaf mice: mouse models for TECTA-based human hereditary deafness reveal domain-specific structural phenotypes in the tectorial membrane. *Hum Mol Genet*. 2014;23(10):2551-2568. doi:10.1093/hmg/ddt646

39. Bullen A, Forge A, Wright A, Richardson GP, Goodyear RJ, Taylor R. Ultrastructural defects in stereocilia and tectorial membrane in aging mouse and human cochleae. *J Neurosci Res*. 2020;98(9):1745-1763. doi:10.1002/jnr.24556

40. Rau A, Legan PK, Richardson GP. Tectorin mRNA expression is spatially and temporally restricted during mouse inner ear development. *J Comp Neurol*. 1999;405(2):271-280.

41. Richardson G, Lukashkin A, Russell I. The tectorial membrane: One slice of a complex cochlear sandwich. *Curr Opin Otolaryngol Head Neck Surg*. 2008;16(5):458-464. doi:10.1097/MOO.0b013e32830e20c4

42. Legan PK, Rau A, Keen JN, Richardson GP. The mouse tectorins. Modular matrix proteins of the inner ear homologous to components of the sperm-egg adhesion system. *J Biol Chem*. 1997;272(13):8791-8801. doi:10.1074/jbc.272.13.8791

43. Alloisio N, Morlé L, Bozon M, et al. Mutation in the zonadhesin-like domain of alpha-tectorin associated with autosomal dominant non-syndromic hearing loss. *Eur J Hum Genet EJHG*. 1999;7(2):255-258. doi:10.1038/sj.ejhg.5200273

44. Strimbu CE, Prasad S, Hakizimana P, Fridberger A. Control of hearing sensitivity by tectorial membrane calcium. *Proc Natl Acad Sci U S A*. 2019;116(12):5756-5764. doi:10.1073/pnas.1805223116

45. Huang RH, Wang Y, Roth R, et al. Assembly of Weibel–Palade body-like tubules from N-terminal domains of von Willebrand factor. *Proc Natl Acad Sci U S A*. 2008;105(2):482-487. doi:10.1073/pnas.0710079105

46. Hildebrand MS, Morín M, Meyer NC, et al. DFNA8/12 caused by TECTA mutations is the most identified subtype of nonsyndromic autosomal dominant hearing loss. *Hum Mutat*. 2011;32(7):825-834. doi:10.1002/humu.21512

47. Yasukawa R, Moteki H, Nishio S ya, et al. The Prevalence and Clinical Characteristics of TECTA-Associated Autosomal Dominant Hearing Loss. *Genes*. 2019;10(10):744. doi:10.3390/genes10100744

48. Balciuniene J, Dahl N, Jalonen P, et al. Alpha-tectorin involvement in hearing disabilities: one gene--two phenotypes. *Hum Genet*. 1999;105(3):211-216. doi:10.1007/s004390051091

49. Naz S, Alasti F, Mowjoodi A, et al. Distinctive audiometric profile associated with DFNB21 alleles of TECTA. *J Med Genet*. 2003;40(5):360-363. doi:10.1136/jmg.40.5.360

50. Nam GS, Rim JH, Choi JY, et al. The TECTA mutation R1890C is identified as one of the causes of genetic hearing loss: a case report. *BMC Med Genet*. 2019;20(1):57. doi:10.1186/s12881-019-0775-1

SUPPORTING TABLES

Supporting Table 1: Summary of the clinical information of familial MD patients carrying variants in the *TECTA* gene.

Families		F1	F2	F3	F4	F5	F6
**Genetic variants**	***TECTA* gene**	p.Val1494Ala	p.Val1494Ala	p.Cys1402Ser	p.Asn1474LysfsTer91	p.Pro1790Ser	p.Gly2118ProfsTer22
**MD patients**	**Index**	III-11	III-6	II-3	II-1	II-3	III-2
	**Other**	II-6 III-7	III-2	I-2	I-2	-	-
**Relatives with incomplete phenotype**	**Episodic vertigo**	II-1, II-8, III-13, III-15, III-19	III-8	-	-	III-1	I-2
	**Hearing loss**	II-3	II-6, III-8	-	-	-	I-2, II-7
**Clinical data of index patient**	**Sex**	Female	Female	Female	Female	Male	Female
	**Laterality (ear)**	Unilateral (Right)	Bilateral	Unilateral (Right)	Bilateral	Bilateral	Unilateral (Left)
	**Age of onset**	41	55	43	22	31	29
	**Autoimmune diseases**	No	No	No	No	No	No
	**Population**	Spanish	Spanish	Spanish	Swiss	Spanish	Spanish

Supporting Table 2: Pair of primers used to validate candidate single nucleotide variants by Sanger Sequencing in the TECTA gene.

Variant (hg38)	Forward primer	Reverse primer
chr11:121152980G>C	TCACCTGCCCTCCAAACAG	CTCGTAATATTTGCCATCGGAGT
chr11:121165368C>T	CCATCTGACCATTTCCAATGTGA	AAAAGGATGTAGCTGTACTTTGAAG
chr11:121158016T>C	ATGCAAGTCAGACGAGGAGT	AAGGAGATGTCGGGCAGTTT

Supporting Table 3: Predicted structural model evaluation of each of the α-tectorin domains, the 4-, 8-, and 10-domain assemblies of the protein using the DEMO method and the α-tectorin structural model predicted by Alphafold2 (located at https://alphafold.ebi.ac.uk/entry/O75443). Molprobity Score, Verify3D, ERRAT, ProSA-web and QMEANDisCo metrics were used in the evaluation. Molprobity Score is a weighted logarithmic combination of different geometric scores such as clashscore, percentage of unfavoured Ramachandran and percentage of bad sidechain rotamers, giving a number that reflects the crystallographic resolution at which those values would be expected. Lower values of Molprobity Score are better. Verify3D determines the compatibility of an atomic model (3D) with its own amino acid sequence (1D) by assigning a structural type based on its location and environment. A higher score indicated high-quality of the structure. The overall quality factor, ERRAT, analyses the statistics of interactions between the different types of atoms and plots the value of the error function calculated by a comparison with highly refined structure statistics. As the generally accepted range for a high- quality model is >50, this analysis revealed that the backbone conformation and nonbonded interactions of all models were within the scope of a high-quality model. In the ProSA-web tool, the score is z-score defined as the energy separation between the native fold and the average of an ensemble of the misfolds in standard deviation units of the database. A z-score outside a range characteristic for native proteins of similar sizes indicated an erroneous structure. In this case, each model is in the range. Finally, QMEANDisCo evaluates the agreement of pairwise distances between residues with sets of distance constraints extracted from structures homologous to the evaluated model, so the higher the score the better the model.

* Selected structural model domain to build the assembly and model the whole protein.

** Model used for evaluating the impact of the variants found in this study.

Protein Domain	Initial domain position	Final domain position	Initial modelled domain position	Final modelled domain position	Modelled length	Modelling method	Template used	Evaluation				
								Molprobity Score	Verify3D	ERRAT	ProSA-Web	QMEANDisCo
**NIDO**	98	252	23	252	230	Modeller	No template available	-	-	-	-	-
						Robetta-AB	-	1.79	85.81	56.0606	-5.57	0.47
						CI-TASSER	-	4.19	98.06	46.25	-5.29	0.34
						AlphaFold2 *	-	1.13	100	87.7729	-5.86	0.68
**VWFC**	260	314	253	314	62	Modeller	No template available	-	-	-	-	-
						Robetta-AB *	-	0.83	96.36	82.6087	-4.94	0.46
						CI-TASSER	-	2.85	38.18	91.4894	-5.16	0.46
						AlphaFold2	-	1.74	71.67	65.1163	-4.65	0.47
**VWFD 1**	320	500	315	500	186	Modeller	7KWO_V	3.2	59.55	47.6415	-3.66	0.55
						AlphaFold2 *	-	1.39	93.84	94.697	-6.36	0.55
**TIL 1**	597	650	501	650	150	Modeller	7A5O_E	2.8	52.23	52.22	-2.87	0.47
						AlphaFold2 *	-	0.5	71.05	92.1348	-4.79	0.63
**VWFD 2**	711	886	651	886	236	Modeller	7KWO_V	3.38	80.18	21.1538	-5.2	0.55
						AlphaFold2 *	-	1.04	99.63	95.2	-6.02	0.6
**TIL 2**	984	1036	887	1036	150	Modeller	7A5O_E	2.8	52.83	52.2727	-2.76	0.46
						AlphaFold2 *	-	0.5	75.44	100	-6.11	0.59
**VWFD 3**	1098	1278	1037	1278	242	Modeller	7KWO_V	3.52	72.6	29.8578	-4.64	0.54
						AlphaFold2 *	-	0.83	97.45	96.3415	-7.33	0.65
**TIL 3**	1372	1425	1279	1425	147	Modeller	7A5O_E	3.12	37.04	13.6364	-2.93	0.49
						AlphaFold2 *	-	0.94	81.42	100	-5.16	0.59
**VWFD 4**	1485	1666	1426	1666	241	Modeller	7KWO_V, 7A5O_E	3.46	95.38	43.6364	-5.58	0.61
						AlphaFold2 *	-	1.18	93.96	89.441	-6.27	0.67
**ZP**	1805	2059	1667	2059	393	Modeller	6ZS5, 4WRN, 6TQL	3.49	73.73	36.3265	-5.31	0.58
						AlphaFold2 *	-	1.61	65.88	94	-9.1	0.53
**Complete α-tectorin (O75443)**	1	2155	1	2155	2155	AlphaFold2	-	1.52	-	89.5459	-17.6	0.57
**4 Domains Assembly**	98	650	23	650	628	AlphaFold2	-	1.47	90.7	83.7879	-9.1	0.6
**8 Domains Assembly**	98	1425	23	1425	1403	AlphaFold2	-	1.13	-	82.1324	-14.93	0.55
**10 Domains Assembly ** **	**98**	**2059**	**98**	**2059**	**1962**	**AlphaFold2**	**-**	**1.05**	**-**	**80.6365**	**-17.29**	**0.55**

Supporting Table 4: Predicted α-tectorin stability perturbation caused by missense variants found in this study.

Predictor	Variants		
	**p.Cys1402Ser**	**p.Val1494Ala**	**p.Pro1790Ser**
Maestro-web (kcal/mol)	Neutral (0.110)	Neutral (0.056)	Neutral (0.022)
mCSM (stability) (kcal/mol)	Destabilizing (-1.254)	Destabilizing (-1.099)	Destabilizing (-0.545)
CUPSAT thermal (kcal/mol)	Destabilizing (-13.02)	Destabilizing (-0.57)	Stabilizing (2.23)
CUPSAT denaturants (kcal/mol)	Stabilizing (12.46)	Stabilizing (0.79)	Neutral (-0.4)
DynaMut2 (kcal/mol)	Destabilizing (-0.54)	Destabilizing (-1.29)	Neutral (-0.25)
PremPS (kcal/mol)	Destabilizing (2.2)	Destabilizing (0.6)	Neutral (0.43)

Global protein stability change prediction (kcal/mol) in the α-tectorin model using different ΔΔGpred prediction methods. For Maestro-web and PremPS, ΔΔGpred < 0.0 indicates a stabilizing mutation. On the other hand, for mCSM, CUPSAT and DynaMut2, ΔΔGpred > 0.0 indicates a stabilizing mutation. Variants were considered neutral in terms of protein stability perturbation when -0.5 < ΔΔGpred < 0.5.

Supporting Table 5: Probable Ca2+ binding sites in the α-tectorin VWFD4 protein structure

Binding site	α-tectorin residue number	Amino acid	Score (MIB)
1	1507	ALA	2.096
	1508	ASN	2.096
2	1530	ASN	1.682
	1531	PHE	1.682
	1532	ASP	1.682
3	1624	ASN	2.143
	1626	ASN	2.143
	1627	GLY	2.099
	1628	ASP	2.143
	1630	THR	1.641
	1631	ASP	2.143
	1632	ASP	2.143
	1499	ASP	1.746
4	1664	SER	2.097
	1666	ASN	2.097

SUPPORTING FIGURES

Supporting Figure 1: Serial pure tone audiograms for MD patients from the six families included in this study carrying variants in the TECTA gene. The different lines in the audiograms show the hearing loss progression across the years (yo). Years after onset are indicated in brackets for each individual.

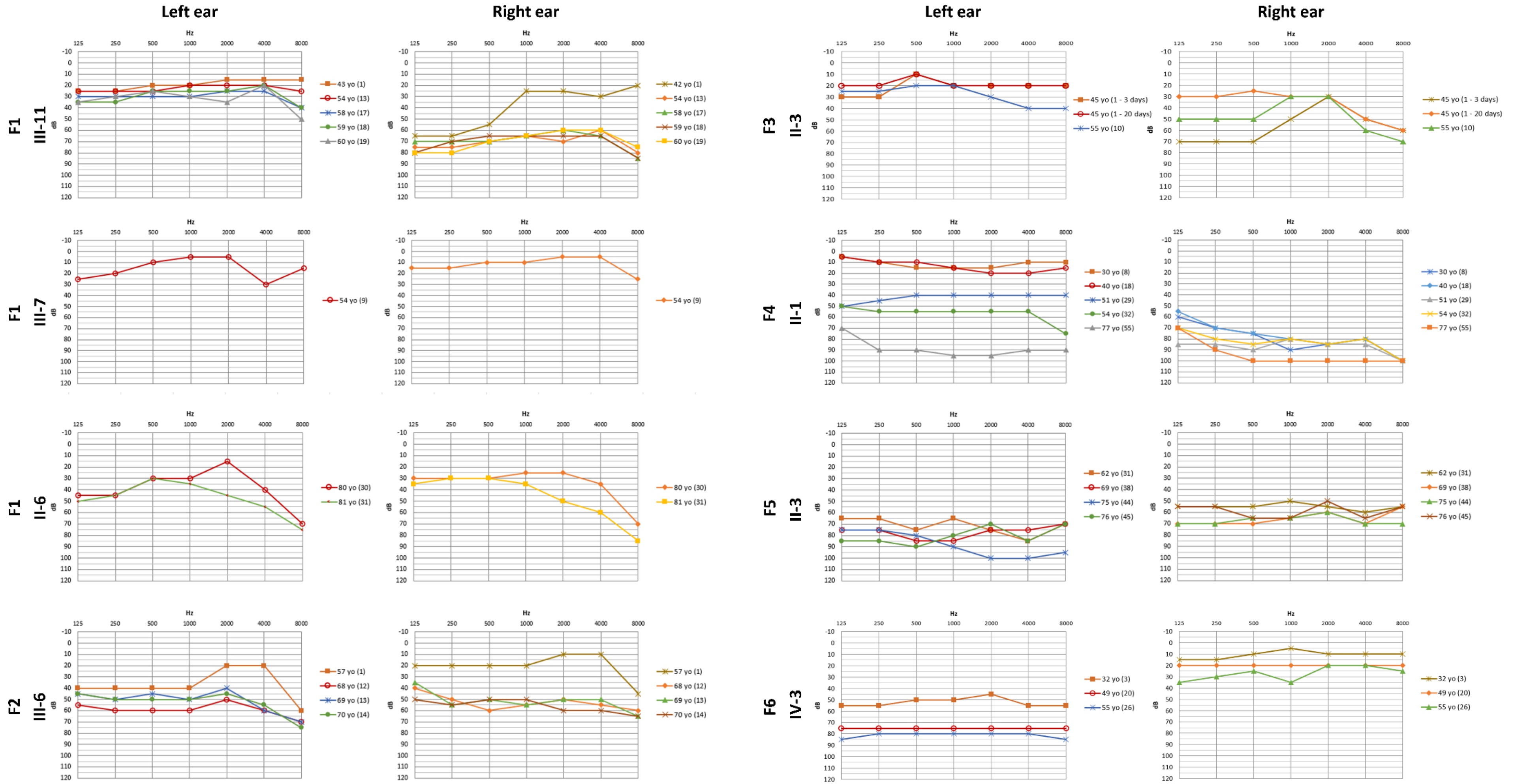

Supporting Figure 2: Validation of candidate variants by Sanger sequencing.

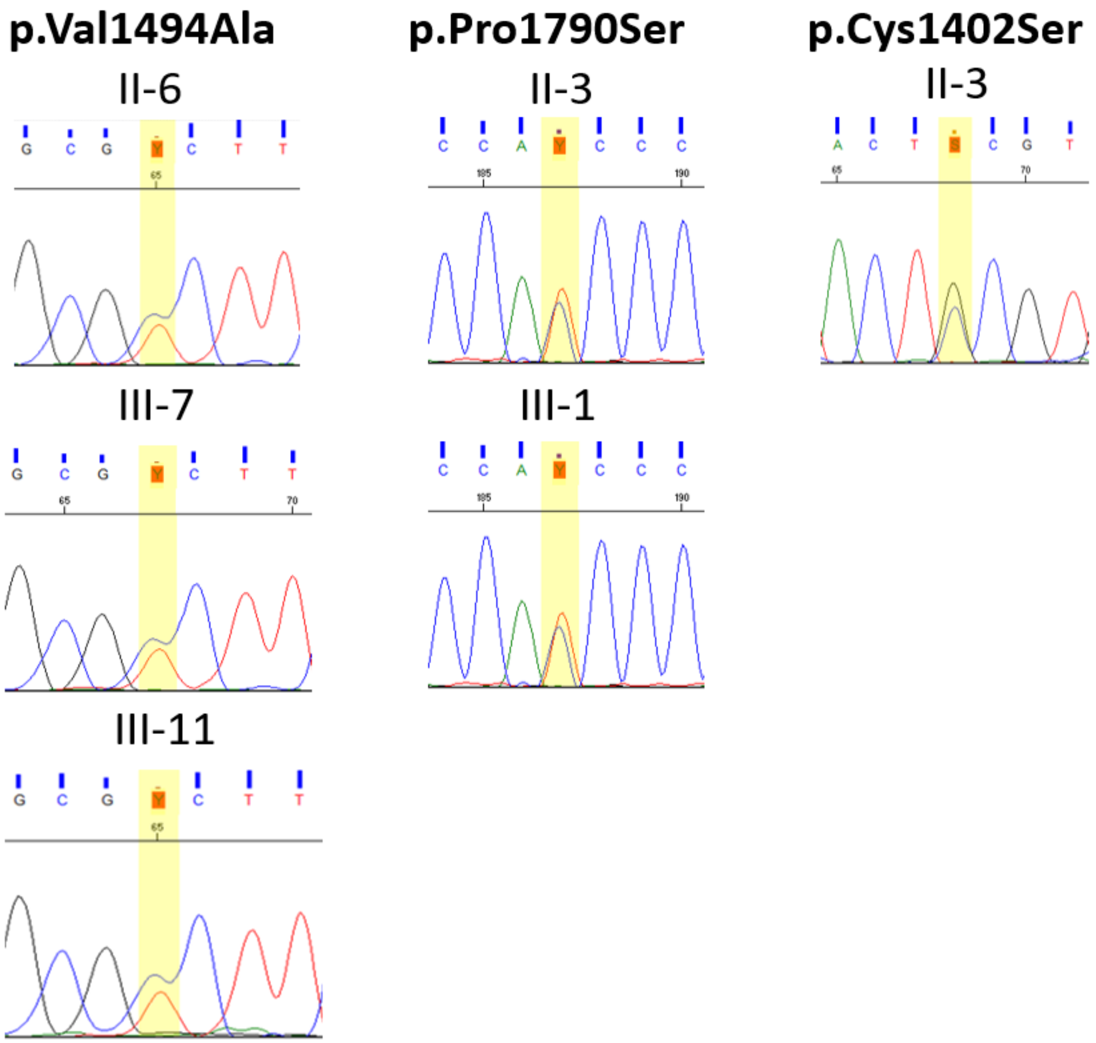

Supporting Figure 3: The four predicted Ca2+ binding sites in the α-tectorin VWFD4 protein structure (Supporting Table 5 – coloured in this figure in green) located near the variant p.Val1494Ala, found in F1, F2 and a sporadic patient with MD.

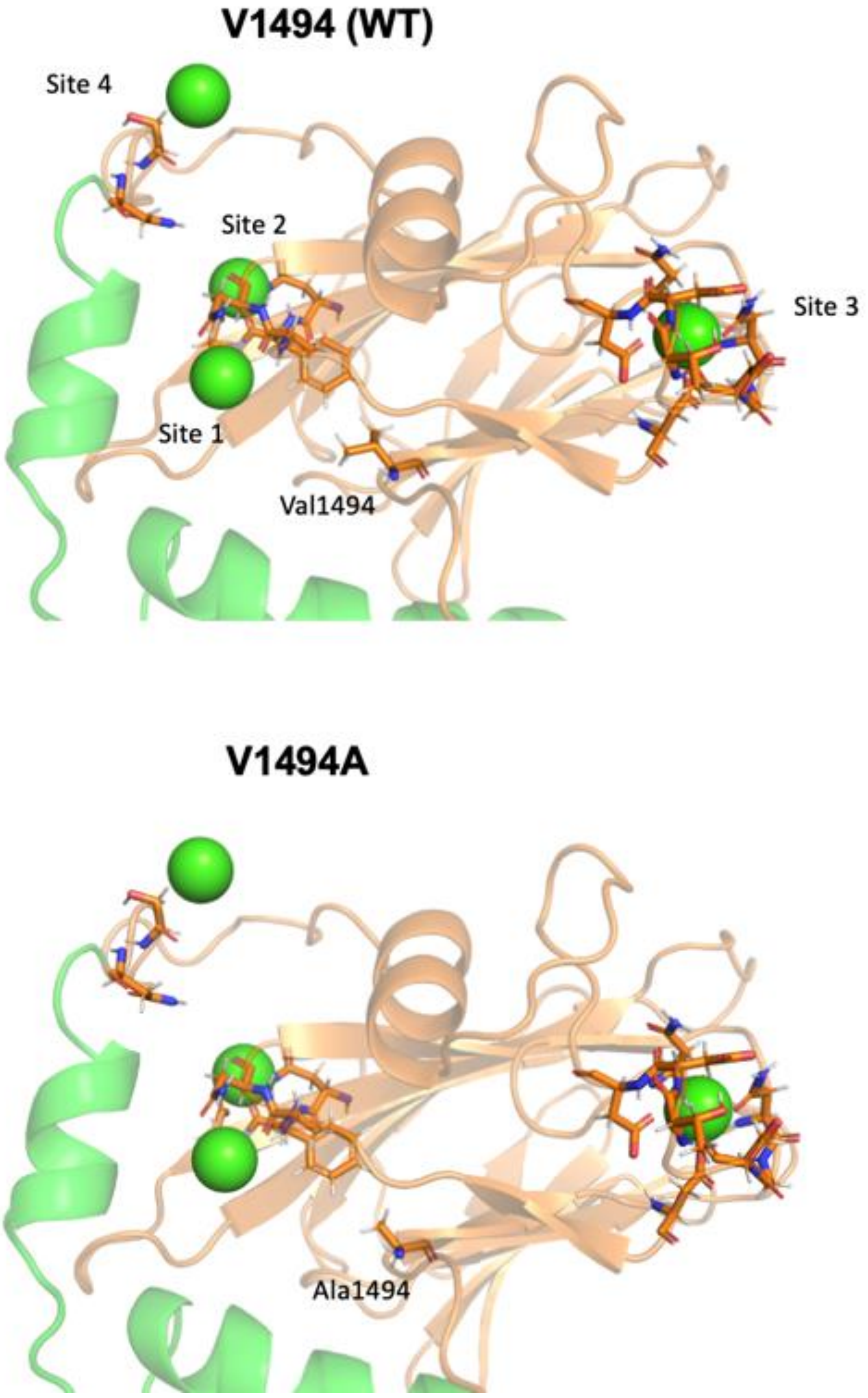

Supplement: Supplementary file 1 — Supporting Information [file CTM2-12-e829-s001.pdf]
